# Clinical improvement may not reflect metabolic homeostasis normalization in subjects with and without Roux-En-Y bariatric surgery after 12 years: comparison of surgical subjects to a lean cohort

**DOI:** 10.3389/fendo.2023.1228853

**Published:** 2023-09-21

**Authors:** Alexandra E. Butler, Manjunath Ramanjaneya, Abu Saleh Md Moin, Steven C. Hunt, Stephen L. Atkin

**Affiliations:** ^1^ Royal College of Surgeons in Ireland Bahrain, Adliya, Bahrain; ^2^ Qatar Metabolic Institute, Hamad Medical Corporation, Doha, Qatar; ^3^ Translational Research Institute, Hamad Medical Corporation, Doha, Qatar; ^4^ Department of Internal Medicine, University of Utah, Salt Lake, UT, United States

**Keywords:** bariatric surgery, adipokines, myokines, adipose tissue, roux-en-Y

## Abstract

**Background:**

A 12-year study comparing clinical outcomes following Roux-en-Y bariatric surgery showed long-term weight loss with remission/prevention of type-2-diabetes (T2D), hypertension and dyslipidemia. However, it is unknown whether the underlying homeostatic metabolic processes involving hepatokines, adipokines and myokines also normalize. Using this 12-year study, we determined whether metabolic indices improved in post-surgical (BMI:34.4kg/m^2^) versus non-surgical comparator-subjects-with-obesity (BMI:43.8kg/m^2^) at 12-year follow-up (both cohorts with baseline diabetes), and if post-surgical subjects normalized their metabolic processes to those of a normal-weight cohort without diabetes.

**Methods:**

Cross-sectional design. Plasma from a cohort of Roux-en-Y bariatric surgery (n=50) and non-surgery (n=76) comparator-subjects-with-obesity (both cohorts at 12-year follow-up) plus a normal-weight cohort (n=39) was assayed by Luminex immunoassay or ELISA for hepatokines [angiopoietin-like proteins-(ANGPTL3; ANGPTL4; ANGPTL6); fibroblast growth factors-(FGF19; FGF21; FGF23)]; adipokines [adipsin; adiponectin; FGF19] and myonectin.

**Results:**

After age and gender adjustment, surgery versus comparator-subjects-with-obesity had lower BMI (34.4 ± 1.0 vs 43.8 ± 0.9kg/m^2^; p<0.0001), HbA1c (6.2 ± 0.3 vs 7.7 ± 0.2%; p<0.0001), insulin resistance (HOMA-IR, 2.0 ± 1.5 vs 10.8 ± 1.4; p<0.0001) fat mass (45.6 ± 2.2 vs 60.0 ± 2.0; p<0.0001), HDL-C (55.4 ± 2.6 vs 42.6 ± 2.3mg/dL; p<0.0001), triglycerides (130 ± 14 vs 187 ± 12mg/dL; p<0.0001) and higher adiponectin (25.9 ± 2.3 vs 15.7 ± 2.0µg/ml; p<0.001); Adipsin, ANGPTL3, ANGPTL4, ANGPTL6, FGF19, FGF21, FGF23 and myonectin did not differ. Surgery versus normal-weight group: higher ANGPTL4 (156 ± 6 vs 119 ± 7ng/mL; p<0.0001), higher FGF23 (96.4 ± 10.1 vs 50.9 ± 11.5pg/mL; p=0.007) and lower myonectin (744 ± 55 vs 969 ± 66ng/mL; p=0.002); adiponectin, adipsin ANGPTL3, ANGPTL6, FGF19, FGF21 did not differ. Non-surgery comparator-subjects-with-obesity versus normal-weight group: higher adipsin (1859 ± 94 vs 1314 ± 133ng/mL; p=0.0001), higher FGF23 (84.6 ± 8.5 vs 50.9 ± 11.5pg/mL; p<0.0001) and higher ANGPTL4 (171 ± 5 vs 119 ± 7ng/mL; p<0.0001); adiponectin ANGPTL3, ANGPTL6, FGF19, FGF21 and myonectin did not differ.

**Conclusion:**

Bariatric surgery markedly improved anthropometric and metabolic features versus comparator-subjects-with-obesity at 12-year follow-up, indicating benefit of weight loss. However, despite weight loss, these patients still had class-1 obesity, as reflected in the adipokine, hepatokine and myokine markers of body homeostasis that did not completely normalize to indicative values of normal-weight subjects, suggesting either that this is the new normal for these patients or that weight loss to a BMI<25kg/m^2^ is needed for normalization of these parameters.

## Introduction

Obesity is a global epidemic that is increasing in prevalence. For those who are severely obese, bariatric surgery is an effective intervention leading to significant and sustained weight loss and improving health-related quality of life, longevity and remission of type 2 diabetes (T2D) (1–3); however, most randomized control trials enroll small numbers of patients with follow up limited to one or two years duration (4) and differing procedures vary in their effectiveness and adverse event rate (5). Whilst many clinical and biochemical parameters improve or normalize following bariatric surgery (1), the question remains as to whether the underlying homeostatic metabolic processes including those of the hepatokine, adipokine and myokine pathways also normalize to those with a normal BMI with relative weight loss, even when weight has not been completely decreased to normal.

Obesity is associated with the development of non-alcoholic fatty liver disease (NAFLD) and the dysregulation of adipokines, hepatokines and myokines that are critical homeostatic pathways. Steatosis, steatohepatitis and fibrosis appear to be improved or completely resolve in subjects after bariatric surgery-induced weight loss (6).

Hepatokines include the fibroblast growth factors (FGFs 19, 21 and 23) that have a vital role in energy metabolism regulation and metabolic homeostasis of bile acids, lipids and glucose (7, 8) that specifically affect obesity and T2D (9). FGF19 is classified as both a hepatokine and an adipokine (10), whilst FGF21 has been reported as a hepatokine, an adipokine and a myokine (11). FGF23 has been associated with insulin resistance and inflammation (12).

Angiopoietin-like proteins (ANGPTLs) are hepatokines, of which ANGPTL3 inhibits lipoprotein lipase (LPL) activity (13) and reduces insulin sensitivity (14), suggesting that ANGPTL3 regulates both lipid and glucose metabolism. ANGPTL4 negatively correlates with circulating triglycerides (15) and is linked with metabolic syndrome and T2D (15), whilst ANGPTL6 is elevated in T2D subjects (16) and correlates with insulin resistance (17).

Adipokines arising from adipose tissue are dysregulated in obesity and T2D. Adiponectin has anti-inflammatory properties (18), decreases the inflammatory response (18) and reduces atherogenesis (19), and may be pivotal in the development of T2D and cardiovascular disease (20–22). Adipsin regulates adipose tissue homeostasis, promoting the accumulation of lipids, and is associated with the development of metabolic disorders (23).

Myonectin is a myokine that has been reported to increase fatty acid uptake in adipocytes and hepatocytes, and its serum concentration negatively correlates with obesity (24).

**Figure 1:**
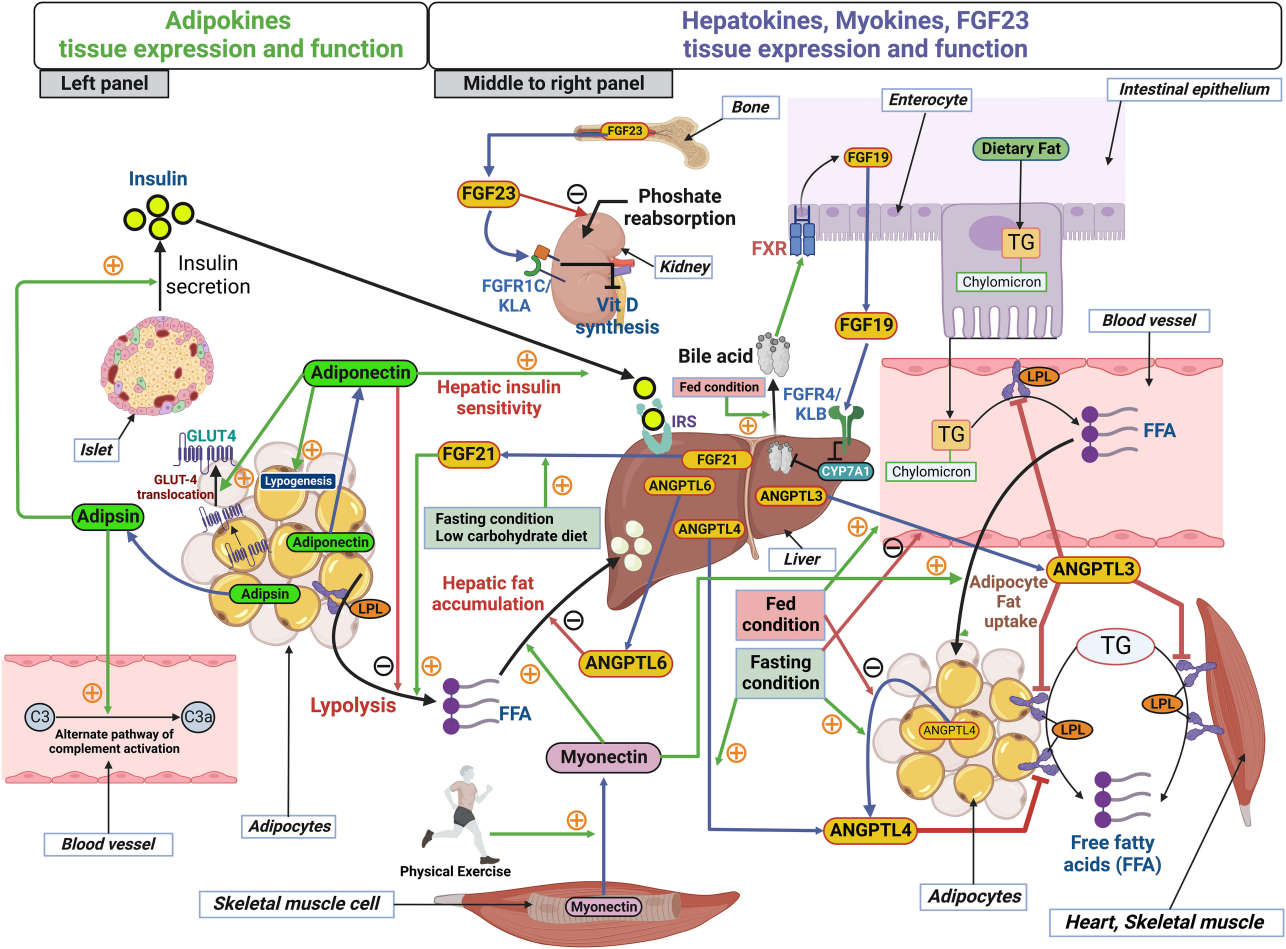
Schematic illustration of the sources of adipokines, hepatokines and myokines and their role in lipid metabolism and energy homeostasis. Left panel, two adipokines, adiponectin and adipsin are released from adipocytes. In adipose tissue, adiponectin enhances GLUT4 translocation and lipogenesis; however, adiponectin inhibits lipolysis in adipose tissue. Adiponectin acts in a paracrine manner in the liver and enhances hepatic insulin sensitivity. Adipsin functions as a modulator of the alternative pathway complement activation. Adipsin enhances pancreatic β-cell function and increase insulin secretion. Middle to right panel, hepatokines, FGF19, FGF21, ANGPTL3, ANGPTL4 and ANGPTL6 are released from hepatocytes and function mainly in a paracrine manner. FGF19 is expressed in the intestinal absorptive cells, enterocytes and is released into the enterohepatic circulation postprandially (fed condition) in response to bile acids via activation of farnesoid X receptor (FXR). FGF19 acts in the liver by activating FGF receptors-4 and co-receptor βKlotho (FGFR4/KLB) complex to provide negative feedback of bile acid synthesis by inactivating the rate limiting enzyme cholesterol 7 alpha-hydroxylase, CYP7A1 in bile acid synthesis. Fasting condition or a low carbohydrate diet induces FGF21 secretion and FGF21 induces lipolysis from adipose tissue. ANGPTL3 release is induced by the fed condition but inhibited by the fasting condition. ANGPTL3 is an inhibitor of lipoprotein lipase (LPL) in endothelial cells in blood vessel, adipocytes and muscle. Thus, ANGPTL3’s action restricts the breakdown of dietary TG-chylomicron and fat from adipocytes and muscle. ANGPTL4 secretion is induced by the fasting condition but inhibited by the fed condition. ANGPTL4 is also released from adipocytes and functions in an autocrine manner to inhibit LPL in adipose tissue. ANGPTL6 inhibits hepatic fat accumulation. Physical exercise increases the secretion of myonectin, the myokine released from skeletal muscle cells. Myonectin enhances fat accumulation in liver and in adipocytes. TG, triglycerides; LPL, Lipoprotein lipase; IRS, Insulin receptor substrate. Secretion of all the molecules from source tissues are indicated by blue arrows. Green arrows with the orange plus symbol indicate activation and red arrows with the black minus symbol indicates inhibition. FGF23 is mainly produced in bone cells, osteoblasts, and osteocytes and acts on the kidney, where it acts on FGF receptor-1c and the co-receptor αKlotho (FGFR1c-KLA complex) in the proximal and distal convoluted renal tubules of kidney. FGF23 inhibits phosphate (Pi) re-uptake and expression of 1α-hydroxylase (CYP27B1), the rate-limiting enzyme for active vitamin D (1α,25(OH)_2_D_3_) production.

Given the many adipokines, hepatokines and myokines, this study focused on those adipokines (25, 26), hepatokines (27–32) and the myokine, myonectin (24), that have been previously reported to be altered in response to bariatric surgery, weight loss, lipid regulation and glucose homeostasis. A schematic illustration of the sources of hepatokines, adipokines and myokines, and their roles in lipid metabolism and energy homeostasis, is shown in **Figure 1**.

At the end of the 12-year period, 53% of the subjects undergoing bariatric surgery included in this study had complete diabetes remission and significantly lower incidence rates of hypertension and dyslipidemia than matched subjects that did not have surgery (1). With the marked improvement of these metabolic features, it was not known if other biochemical indices (such as adipokines, hepatokines and myokines) would remain unchanged due to the surgical group still having class 1 obesity, show an incremental decrease with weight loss, or return to the levels of those of a normal BMI population (<25kg/m^2^). Therefore, this study determined the levels of hepatokine, adipokine and myokine pathway homeostasis parameters 12 years after Roux-en-Y surgery in this well characterized longitudinal cohort (1) compared to comparator subjects with similar obesity at baseline who did not have surgery and compared these to a normal-weight cohort.

## Materials and methods

This study was performed on frozen samples from subjects participating in a 12-year follow-up exam of a well-characterized longitudinal cohort with and without bariatric surgery (1), with a comparison to an additional group of normal-weight subjects. From February 1, 2010, to June 16, 2011, subjects seeking bariatric surgery at the Rocky Mountain Associated Physicians surgical center, Salt Lake City, were recruited for participation in this study and gave their written informed consent to participate (1). Roux-en-Y gastric bypass (RYGB) surgery was performed by three surgeons (partners) using the same surgical technique. From this cohort, 50 subjects who had RYGB and 76 subjects who sought RYBG but ultimately did not undergo surgery, all with T2D at baseline, were selected for additional biochemical measurements. Baseline demographic data has previously been reported (1). Subjects at baseline had severe obesity (BMI 35 to 60 kg/m^2^), were aged 25–60 years, of either gender, of all races, had no active cancer, history of myocardial infarction, coronary bypass surgery, percutaneous transluminal coronary angioplasty (PTCA) or stroke and had a fasting blood sample available from a 12-year follow-up examination (**Table 1**). Fat mass was determined by bioelectrical impedance (RJL Systems Analyzer, Quantum II, Clinton, MI, USA). Biochemical measurements were made on the 12-year follow-up samples kept at -80^0^C after collection. Treated type 2 diabetes was defined by current use of T2D prescription medications. None of the subjects were on weight reducing medication.

**Table 1:** Demographics of the study population: surgery and non-surgery with obesity at 12 years and the normal BMI group.

Variable	Surgery (n=50)	Non-surgery comparator subjects with obesity (n=76)	Normal-weight subjects (n=39)	ANOVA
	Mean ± SD	Mean ± SD	Mean ± SD	p-value
% Female	76	76	67	
age (y)	59.0 ± 10.5	62.7 ± 9.4	48.5 ± 6.4^###^	<0.0001
BMI (kg/m^2^)	34.3 ± 7.9	43.6 ± 7.5^###^	23.6 ± 1.8^###^	<0.0001
Insulin (uIU/ml)	10.3 ± 8.2	34.3 ± 48.4^###^	7.5 ± 3.9	<0.0001
Fat free mass (kg)	50.7 ± 14.3	56.9 ± 13.1^#^	48.4 ± 10.4	0.003
Fat mass (kg)	46.6 ± 15.8	60.2 ± 15.4^###^	18.8 ± 5.9^###^	<0.0001
HOMA-β	120 ± 88	130 ± 129	124 ± 75	0.88
HOMA-IR	2.7 ± 2.5	11.5 ± 14.8^###^	1.6 ± 0.9	<0.0001
Glucose (mg/dL)	103.1 ± 42.4	142.6 ± 57.2^###^	86.3 ± 6.0	<0.0001
HbA1c (%)	6.4 ± 1.4	7.9 ± 1.8^###^	NA	<0.0001
TG (mg/dl)	122 ± 63	172 ± 112^#^	114 ± 89	0.0015
LDL-C (mg/dl)	101 ± 30	104 ± 36	123 ± 30^#^	0.003
HDL-C (mg/dl)	58 ± 22	47 ± 14^#^	56 ± 18	0.0007
AST (U/L)	22 ± 9	25 ± 18	NA	0.33
ALT (U/L)	21 ± 12	26 ± 17	NA	0.054
FGF19 (pg/ml)	413± 340	218 ± 161	334 ± 368	0.09
FGF21 (pg/ml)	259± 248	239 ± 241	185 ± 225	0.36
FGF23 (pg/ml)	98.7 ± 67.7	83.2 ± 64.1	60.8 ± 57.9^#^	0.026
ANGPTL3 (ng/ml)	12.8 ± 4.3	11.8 ± 4.0	13.0 ± 4.1	0.26
ANGPTL4 (ng/ml)	161 ± 46	183 ± 48^#^	103 ± 27^###^	<0.0001
ANGPTL6 (ng/ml)	1997 ± 945	2359 ± 1140	1533 ± 889	0.0004
Adipsin (ng/ml)	1619 ± 683	1922. ± 840	1111 ± 372^#^	<0.0001
Adiponectin (µg/ml)	27.1 ± 17.9	18.1 ± 14.0^#^	21.6 ± 13.6	0.007
Myonectin (ng/ml)	758 ± 322	835 ± 407	1004 ± 327^#^	0.007

BMI, body mass index; HbA1c, glycated hemoglobin; HOMA-β, homeostatic model assessment of beta cell function; HOMA-IR, homeostatic model assessment of insulin resistance; ALT, alanine aminotransferase; AST, aspartate aminotransferase; LDL-C, low density lipoprotein cholesterol; HDL-C, high density lipoprotein cholesterol; TG, triglycerides; FGF, fibroblast growth factor; ANGPTL, angiopoietin-like protein. Pairwise tests significant at: # p<0.017; ### p<0.0001 nonsurgical comparator subjects with obesity or normal-weight subjects versus the surgery group after adjustment for three post-hoc tests from ANOVA analysis. Unadjusted means and standard deviations.

**Figure 2:**
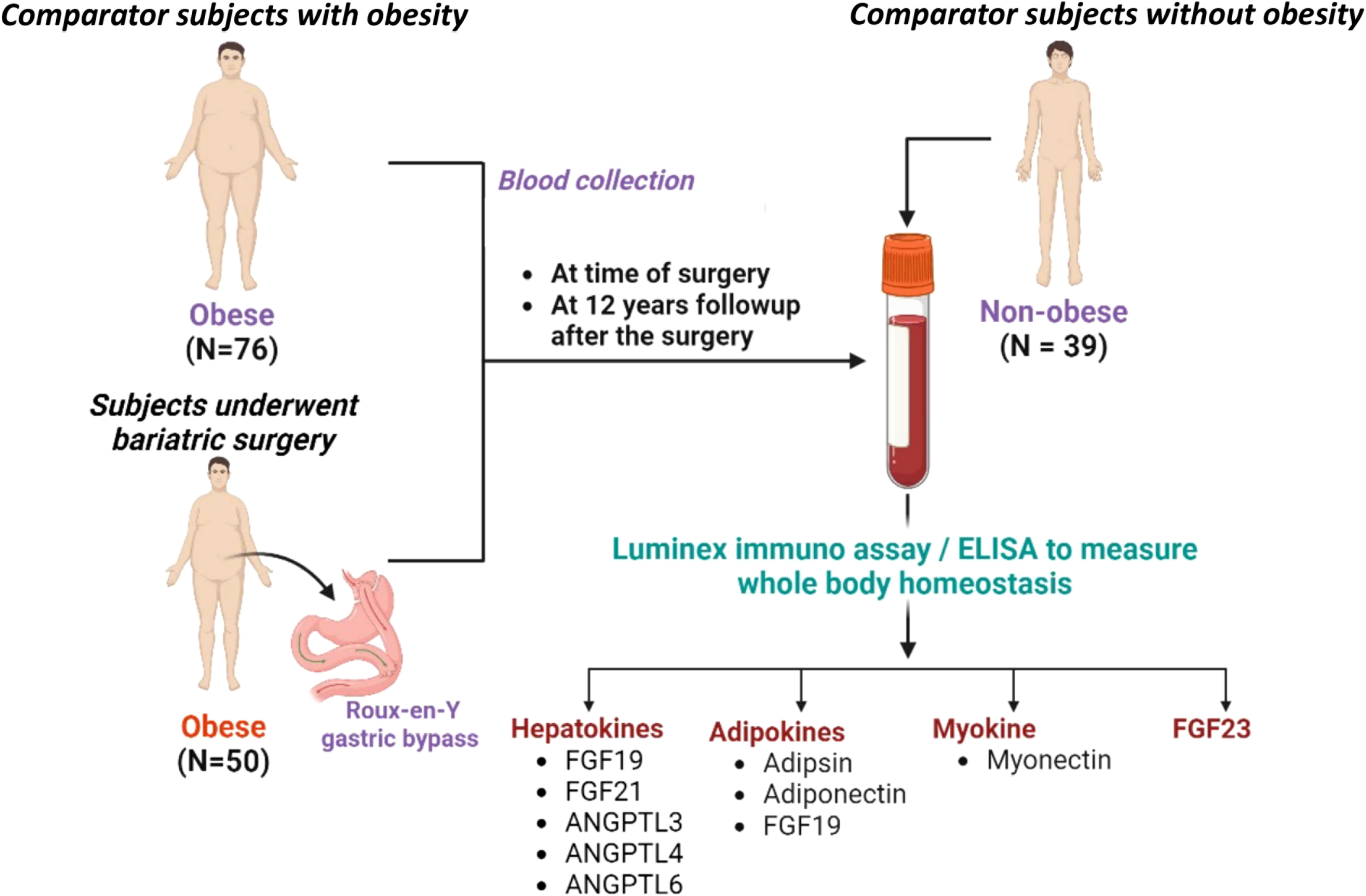
Outline of the study examining the effect of gastric bypass surgery on plasma hepatokines, adipokines, and myokines. The study subjects were subjects with diabetes who underwent bariatric surgery (n=50) compared to subjects with diabetes who were obese and did not have surgery (n=76), both having blood withdrawn 12 years after the baseline exam. Blood from the baseline exam was not analyzed. Surgical subjects were also compared to a normal-weight cohort (n=39). Frozen stored blood samples were used to measure plasma hepatokines [FGF21, angiopoietin like 3 (ANGPTL3), angiopoietin like 4 (ANGPTL4) and angiopoietin like 6 (ANGPTL6)]; adipokines (adiponectin, adipsin, measured by Luminex immune assay; and the myokine,myonectin, measured by ELISA.

The normal-weight group of 39 subjects was selected from a study of hormone and electrolyte responses to saline infusion using plasma from fasting baseline measurements of normotensive individuals without diabetes. Mean BMI in this group was less than 25 kg/m^2^ (**Table 1**). Both studies were approved by the University of Utah’s Institutional Review Board. The use of samples for follow up studies was explicit in the written consent. A schematic outline of the study is shown in **Figure 2**.

### Biochemical analysis

Standard clinical chemistries were obtained from the University of Utah clinical laboratory (glucose, ALT and AST). Insulin was measured by RIA kits according to the manufacturer’s instructions.

### Protein measurement

A custom designed assay from MILLIPLEX MAP Human Liver Protein Magnetic Bead Panel - Metabolism Multiplex Assay Kit (Merck Millipore, USA, Catalog number #HLPPMAG-57K), which is a magnetic bead-based assay, was used to measure levels of FGF19, FGF21, FGF23, ANGPTL3, ANGPTL4, and ANGPTL6 in the plasma samples according to the manufacturer’s instructions. Each plasma sample was diluted 2 times with assay buffer for protein measurement as per the manufacturer’s instructions. Detection range for FGF19 was 100 – 0.14 ng/ml and the intra-assay coefficient of variation (CV) was<3.3% and the inter-assay CV was <12%. Detection range for FGF21 was 10 – 0.01 ng/ml and the intra-assay CV was <1.5% and inter-assay CV <12.4%. The detection range for FGF23 was 30 – 0.04 ng/ml and the intra-assay CV was <2.0% and the inter-assay CV <14.8%.

The detection range for ANGPTL3 was 500 – 0.69 ng/ml and the intra-assay CV was <2.4% and inter-assay CV <7.5%. The detection range for ANGPTL4 was 4000 – 5.49 ng/ml and the intra-assay CV was <2.3% and inter-assay CV <13.7%. The detection range for ANGPTL6 was 1000 – 1.37 ng/ml and the intra-assay CV was <2.5% and inter-assay CV <14.7%. Five-parameter logistic regression algorithms built into the Bio-Plex Manager 6.0 software were used to assess levels of protein levels in reference to standards. Analysis was conducted using a Bioplex-200 instrument according to the manufacturer’s instructions (BIO-RAD, Hertfordshire, UK).

### Adiponectin and adipsin measurement

Adiponectin and adipsin were measured using Bio-Plex Pro Human Diabetes Adipsin and Adiponectin Assays (Bio-Rad Laboratories Ltd, Hertfordshire, U.K, Catalog number #171A7002M). Plasma samples were diluted 380 times with sample dilution buffer for adiponectin and adipsin measurement as per the manufacturer’s instructions. Coefficient of variation and minimal detection levels for adiponectin was CV < 7.8% and minimal detection level 6.9 pg/ml and for adipsin was CV <11.8% and minimal detection level 9.3 pg/ml. Five-parameter logistic regression algorithms built into the Bio-Plex Manager 6.0 software were used to assess levels of adiponectin and adipsin with reference to standards. Analysis was conducted using a Bioplex-200 instrument according to the manufacturer’s instructions (BIO-RAD, Hertfordshire, UK).

### Myonectin (CTRP15) measurement

Plasma myonectin levels were measured using a commercially available ELISA kit (AVISCERA BIOSCIENCE, INC. CA, USA; Catalog number # SK00393-10). Plasma samples were diluted 4 times with sample dilution buffer for myonectin measurement as per manufacturer’s recommended protocol. This assay has a coefficient of variation of <12% and minimum detection limit of 1 ng/ml.

### Statistical analysis

Data points are represented as means ± SEM unless otherwise stated. Because of some very high values for some proteins, the variables were winsorized only at the upper 95% percentile to make the distributions more normal. Statistical analysis was performed using SAS statistical software (SAS version 9.4, Cary, NC). The differences in mean plasma concentrations between surgery, non-surgery comparator-subjects-with-obesity and normal-weight subjects were determined by one-way ANOVA both before and after adjusting for age and gender followed by 3 *post-hoc* tests with Bonferroni correction (p<0.017) for testing the three pairwise comparisons. Use of antidiabetic medication at the 12-year follow-up was included as a covariate in additional models since medications may influence the lab measurements. Because some variables were still not normally distributed after winsorization, the analyses were repeated using nonparametric Kruskal-Wallis tests followed by pairwise comparisons using the Dwass, Steel, Critchlow-Fligner method. For measurements such as HbA1c, AST and ALT where data from normal-weight subjects were missing, these measurements were analyzed by t-test. Group comparison data is shown as actual p value with significance determined from the Bonferroni-corrected p values.

## Results

Baseline weight in the surgical group was mean 133.9kg (131.6-136.3 95% CI) and weight at 12-years was mean 97.5kg (94.7-99.2 95% CI) (-26.9% change from baseline weight) (1). Baseline weight in the non-surgical comparator-subjects-with-obesity was mean 129.4kg (127.4-132.2 95% CI) and weight at 12-years was mean 122.9kg (119.2-126.7 95% CI) (-2% change from baseline weight) (1).

12-year follow-up data with unadjusted means and standard deviations for the post-bypass surgery subjects (n=50), the non-surgery comparator-subjects-with-obesity (n=76), and the normal-weight cohort (n=39) are shown in **Table 1**. **Table 2** shows the p-values from the overall ANOVA and Kruskal-Wallis tests and the pairwise p-values for all three groups for each method. The only difference between the two analytical methods in declaring a significant result was for the surgery versus non-surgery comparator-subjects-with-obesity comparison of ANGPLT4, as the non-parametric p-value no longer met the adjusted p<0.017 threshold for significance. Further analysis used only parametric methods.

**Table 2:** Comparisons of p-values from parametric ANOVA significance tests to nonparametric Kruskal-Wallis tests.

Variable	ANOVA	S vs O	S vs N	O vs N	Kruskal-Wallis	S vs O	S vs N	O vs N
Insulin	<0.0001	<0.0001	0.69	<0.0001	<0.0001	<0.0001	0.10	<0.0001
HOMA-β	0.88	0.61	0.87	0.77	0.68	0.97	0.87	0.62
HOMA-IR	<0.0001	<0.0001	0.61	<0.0001	<0.0001	<0.0001	0.020	<0.0001
Glucose	<0.0001	<0.0001	0.085	<0.0001	<0.0001	<0.0001	0.038	<0.0001
HbA1c	<0.0001	<0.0001	N/A	N/A	<0.0001	<0.0001	N/A	N/A
TG	0.0015	0.004	0.68	0.002	<0.0001	0.003	0.42	<0.0001
LDL-C	0.003	0.71	0.002	0.003	0.0023	0.99	0.004	0.006
HDL-C	0.0007	0.0004	0.55	0.008	0.0005	0.0019	0.95	0.007
AST	0.33	0.33	N/A	N/A	0.91	0.91	N/A	N/A
ALT	0.054	0.054	N/A	N/A	0.088	0.088	N/A	N/A
FGF19	0.089	0.028	0.36	0.22	0.045	0.037	0.26	0.83
FGF21	0.36	0.65	0.16	0.27	0.10	0.84	0.10	0.18
FGF23	0.026	0.20	0.007	0.076	0.005	0.39	0.006	0.032
ANGPTL3	0.26	0.19	0.89	0.17	0.21	0.30	0.99	0.32
ANGPTL4	<0.0001	0.008	<0.0001	<0.0001	<0.0001	0.042	<0.0001	<0.0001
ANGPTL6	0.0004	0.061	0.039	<0.0001	0.0011	0.38	0.07	0.0007
Adipsin	<0.0001	0.023	0.0016	<0.0001	<0.0001	0.18	0.0012	<0.0001
Adiponectin	0.007	0.0016	0.11	0.26	0.011	0.012	0.52	0.20
Myonectin	0.007	0.25	0.0019	0.020	0.0003	0.64	0.0002	0.004

S, Post-surgery group; O, Non-surgery comparator-subjects-with-obesity; N, group with normal-weight subjects; ANOVA, analysis of variance; BMI, body mass index; HbA1c, glycated hemoglobin; HOMA-β, homeostatic model assessment of beta cell function; HOMA-IR, homeostatic model assessment of insulin resistance; ALT, alanine aminotransferase; AST, aspartate aminotransferase; LDL-C, low density lipoprotein cholesterol; HDL-C, high density lipoprotein cholesterol; TG, triglycerides; FGF, fibroblast growth factor; ANGPTL, angiopoietin-like protein. The three pairwise comparisons of the three groups are shown for both the parametric and non-parametric analyses.

N/A: the variable was not measured in the normal-weight group.

### Bariatric surgery versus non-surgical comparator subjects with obesity

Type 2 diabetes status at the 12-year post-surgery follow up timepoint showed that 26 of 50 (52%) subjects had diabetes remission (HbA1c <6.5% and no anti-diabetes medication) whilst all 76 (100%) of the comparator-subjects-with-obesity had T2D (i.e., no remission). The number of subjects receiving anti-diabetes medication at the 12-year post-surgery was 22 of 50 (44%) whilst for the comparator-subjects-with-obesity it was 72 of 76 (95%). Inclusion of antidiabetic medication use as a covariate in the above models did not change the conclusions, as the covariate was not significant in any model. Significantly lower incidence rates of hypertension and dyslipidemia, p<0.05, were also reported (1).

After adjustment for gender and age, the non-surgical comparator-subjects-with-obesity had a higher BMI (27% difference) and the normal-weight cohort had a lower BMI (31% difference) compared to the post-surgery subjects; bariatric surgery did not reduce BMI to normal-weight levels (**Table 3**). Fasting blood glucose was higher in the comparator-subjects-with-obesity (38% difference) and lower in the normal-weight group (16% difference), in accordance with the follow-up BMI differences and the fact that some, but not all, diabetes was resolved in the post-surgery group. Relative to surgery, in the comparator-subjects-with-obesity, insulin (233% difference), triglycerides (TG, 41% difference), HOMA-IR (326% difference), fat mass (29% difference) and fat free mass (12% difference) were elevated, whilst high density lipoprotein cholesterol (HDL-C; 19% difference) was lower. For the parameters of homeostasis, higher adiponectin (33% difference) alone differed whilst adipsin, ANGPTL3, ANGPTL4, FGF19, FGF21, FGF23 and myonectin did not differ.

**Table 3:** Demographics of the study population: surgery and non-surgery with obesity at 12 years and the normal BMI group.

Variable	Surgery	Non-surgery comparator- subjects- with-obesity	Normal-weight subjects	Effect Sizes			ANCOVA p
	Mean ± SEM	Mean ± SEM	Mean ± SEM	Surgery vs Non-surgery comparator-subjects-with- obesity	Surgery vs Normal-weight	Non-surgery comparator-subjects-with- obesity vs Normal-weight	
BMI (kg/m^2^)	34.4 ± 1.0	43.8 ± 0.9	23.4 ± 1.2	-9.4 ± 1.3^###^	11.1 ± 1.6^###^	20.5 ± 1.6^###^	<0.0001
Glucose (mg/dL)	105 ± 7	146 ± 6	84 ± 8 84 ± 8	-41 ± 8^###^	21 ± 11	62 ± 11^###^	<0.0001
HbA1c (%)	6.2 ± 0.3	7.7 ± 0.2	NA	-1.5 ± 0.3^###^	NA	NA	<0.0001
Insulin (uIU/ml)	7.4 ± 5.0	30.6 ± 4.4	7.9 ± 6.0	-23.2 ± 6^##^	-0.5 ± 7.8	22.6 ± 7.8^#^	0.0004
AST (U/L)	20.4 ± 2.3	26.3 ± 2.0	NA	-2.6 ± 2.8	NA	NA	0.36
ALT (U/L)	22.0 ± 2.3	24.6 ± 2.0	NA	-5.9 ± 2.8	NA	NA	0.04
TG (mg/dl)	130 ± 14	187 ± 12	98 ± 17	-57 ± 17^#^	32 ± 22	89 ± 22^###^	<0.0001
LDL-C (mg/dl)	98.6 ± 4.8	103.6 ± 4.2	113.7 ± 5.8	-5.1 ± 5.9	-15.1 ± 7.5	-10.0 ± 7.5	0.13
HDL-C (mg/dl)	55.4 ± 2.6	42.6 ± 2.3	57.1 ± 3.1	12.8 ± 3.2^###^	-1.7 ± 4.0	-14.5 ± 4.1^#^	<0.0001
HOMA-IR	2.0 ± 1.5	10.8 ± 1.4	1.5 ± 1.8	-8.8 ± 1.9^###^	0.9 ± 2.4	9.7 ± 2.4^###^	<0.0001
Fat free mass (kg)	56.4 ± 1.5	63.3 ± 1.4	50.0 ± 1.7	-7.0 ± 1.9^###^	6.3 ± 2.3^##^	13.3 ± 2.3^###^	<0.0001
Fat mass (kg)	45.6 ± 2.2	60.0 ± 2.0	16.0 ± 2.5	-14.4 ± 2.7^###^	29.5 ± 3.3^###^	43.9 ± 3.3^###^	<0.0001
HOMA-β	113 ± 16	125 ± 15	116 ± 19	-11 ± 20	-3 ± 25	9 ± 25	0.84
FGF19 (pg/ml)	400 ± 60	192 ± 79	362 ± 80	209 ± 91	39 ± 105	-170 ± 125	0.08
FGF21 (pg/ml)	244 ± 37	207 ± 32	241 ± 44	37.6 ± 44.5	2.8 ± 57.7	-34.8 ± 56.7	0.66
FGF23 (pg/ml)	96.4± 10.1	84.6 ± 8.5	50.9 ± 11.5	11.9 ± 12.1	45.6 ± 15.3^##^	33.7 ± 15.0	0.013
ANGPTL3 (ng/ml)	12.6 ± 0.6	11.5 ± 0.6	13.2 ± 0.6	1.1 ± 0.8	-0.6 ± 1.0	-1.7 ± 1.0	0.16
ANGPTL4 (ng/ml)	156 ± 6	171 ± 5	119 ± 7	-16 ± 8	37 ± 10^##^	52.2 ± 9.6^###^	<0.0001
ANGPTL6 (ng/ml)	2007 ± 160	2390 ± 139	1473 ± 187	-11 ± 22	34 ± 32	917≠245^##^	0.0010
Adiponectin (µg/ml)	25.9 ± 2.3	15.7 ± 2.0	24.8 ± 2.9	10.3 ± 2.8^##^	1.1 ± 3.6	-9.1 ± 3.6^#^	0.0008
Adipsin (ng/ml)	1617 ± 106	1859 ± 94	1314 ± 133	-242 ± 130	302 ± 169	545 ± 169^#^	0.005
Myonectin (ng/ml)	744 ± 55	830 ± 49	969 ± 66	-86 ± 67	-225 ± 86^#^	-139 ± 86	0.03

ANCOVA, analysis of covariance; BMI, body mass index; HbA1c, glycated hemoglobin; HOMA-β, homeostatic model assessment of beta cell function; HOMA-IR, homeostatic model assessment of insulin resistance; ALT, alanine aminotransferase; AST, aspartate aminotransferase; LDL-C, low density lipoprotein cholesterol; HDL-C, high density lipoprotein cholesterol; TG, triglycerides; FGF, fibroblast growth factor; ANGPTL, angiopoietin-like protein. Pairwise tests significant at: # p<0.017; ## p<0.001; ### p<0.0001 nonsurgical comparator subjects with obesity or normal-weight subjects versus the surgery group after adjustment for three post-hoc tests from ANCOVA analysis.

Age and Gender-adjusted Means, Standard Errors and Effect Sizes.

### Bariatric surgery versus normal-weight comparator subjects

Surgery versus normal-weight group: higher ANGPTL4 (24% difference), higher FGF23 (47% difference), and lower myonectin (30% difference) were seen; adiponectin, ANGPTL3, FGF19 and FGF21 did not differ.

### Non-surgical comparator-subjects-with-obesity versus normal-weight subjects

Non-surgery comparator-subjects-with-obesity versus normal weight group: higher adipsin (29% difference), higher FGF23 (40% difference), higher ANGPTL4 (30% difference) and lower (17% difference) were seen; adiponectin, ANGPTL3, FGF19, FGF21 and myonectin did not differ.

Only HDL-C and fat free mass differences among groups were significantly related to sex, but sex was kept in all ANCOVA models to be consistent.

## Discussion

This study cohort reported marked improvement in many of the anthropometric and metabolic features with weight loss following bariatric surgery compared with the non-surgical comparator-subjects-with-obesity, including the remission of T2D (1). However, absolute weight was still elevated in the post-surgery group (mean BMI 34 kg/m^2^) along with increased free fat mass and glucose levels. This was reflected in the parameters of hepatokines, adipokines and myokines, reflecting body homeostasis that showed that many remained significantly different between both surgery/non-surgical subjects and normal-weight groups, and did not differ between the surgery compared to the non-surgery comparator-subjects-with-obesity (mean BMI 43.8 kg/m^2^). This shows that whilst weight loss has a beneficial and positive effect on clinical parameters such as the resolution of T2D, the body homeostasis parameters were still abnormal compared to normal-weight subjects, suggesting that either this is the new normal threshold for these parameters, or that absolute weight reduction to less than 34 kg/m^2^ (and perhaps even to a BMI of 25 kg/m^2^ and less to be in the normal range) are needed to achieve non-obese levels of these proteins. Whilst this outcome may have been predicted, with the surgical group having such a marked improvement in diabetes remission and lower incidence rates of hypertension and dyslipidemia (1), this study needed to be performed to confirm this outcome for the homeostasis parameters.

Despite the marked weight loss following surgery, hepatokine, adipokine and myokine parameters did not differ to the non-surgery comparator-subjects-with-obesity apart from adiponectin that increased significantly after surgery; this result suggests that adiponectin is more sensitive to weight changes, and that weight loss, but not necessarily to the level of a BMI less than 34 kg/m^2^, may reset the homeostatic set point of adiponectin. Adiponectin was lower in the non-surgical comparator-subjects-with-obesity compared to normal-weight subjects. The increase in adiponectin following surgery is well recognized (33), with levels returning back to those of comparator-subjects-with-obesity (34). Normalization of adiponectin in animal models has been associated with preventing cardiac dysfunction (35) indicating the importance of its restoration to non-obese levels. Adipsin, the other adipokine measured, was higher in the non-surgical comparator-subjects-with-obesity compared to normal-weight subjects though was no different to the surgical patients; adipsin was not shown to change after weight loss following sleeve gastrectomy (36) indicating that weight loss, but not to normal weight, was ineffective for modulation of this adipokine, as is confirmed here.FGF23 levels were significantly lower in normal-weight subjects compared to surgery and the non-surgical comparator-subjects-with-obesity, an observation that has been reported following sleeve gastrectomy (30). Whilst FGF23 is recognized to decrease after surgery (30), that fall was not compared to a normal population and therefore a novel finding of this study is that the fall reported here is not to the level found in subjects with a normal BMI. This is perhaps not surprising as FGF23 has been associated with insulin resistance and inflammation (12) and, whilst there was a marked decrease in body weight after surgery that would be associated with a reduction in both insulin resistance and inflammation, these patients still had a significant weight difference compared to normal-weight subjects.

Similarly, the ANGPTL4 hepatokine levels were significantly lower in normal-weight subjects compared to surgery patients, whilst they did not differ between surgery and comparator-subjects-with-obesity. ANGPTL4 is recognized to fall following Roux-en-Y surgery (31, 32); however, that fall was not compared to a normal population and therefore a novel finding of this study is that the fall reported here is not to the level found in subjects with a normal BMI. This again suggests that restoration of the homeostatic parameters may only occur with weight loss to below a BMI of 34 kg/m^2^ or perhaps even to a normal BMI. Given that ANGPTL4 is recognized to have roles in glucose and lipoprotein metabolism (15, 17, 37), whilst there was a marked decrease in body weight after surgery associated with a decrease in glucose, those glucose levels were still higher than the subjects with normal BMI and hence ANGPTL4 had not fallen to normal levels after surgery.

The hepatokine, ANGPTL6, did not differ between groups. The ANGPTL6 knockout mice model of ANGPTL6 leads to central obesity, insulin resistance and deposition of fat in the liver and skeletal muscle. Circulating levels of ANGPTL6 are elevated in T2D subjects (16) and correlate with insulin resistance (17); therefore, it was surprising that there was no difference between surgical and non-surgical comparator-subjects-with-obesity; however, this may have been a result of not achieving a lower BMI in the surgical group.

The hepatokine ANGPTL3 did not differ between surgery and comparator-subjects-with-obesity or normal-weight subjects; ANGPTL3 levels have been shown to differ post bariatric surgery depending on the type of surgery undertaken though, in accord with the results here, they were reported not to change following Roux-en-Y surgery (31), suggesting that differing bariatric surgery procedures have differing metabolic impacts.

Myonectin was higher in the normal-weight subjects compared to surgery and non-surgery comparator-subjects-with-obesity, and did not differ between surgery and non-surgical comparator-subjects-with-obesity suggesting that the weight loss achieved was insufficient to return this myokine to normal values. Myonectin regulates local and systemic lipid metabolism and myonectin-knockout male mice had significantly elevated VLDL–triglycerides and strikingly impaired lipid clearance from the circulation following an oral lipid load; fat distribution between adipose and liver was also altered in myonectin-deficient male mice fed a high fat diet (38).

Serum myonectin concentration negatively correlated with obesity and it is reported to increase following laparoscopic sleeve gastrectomy (24), though here it did not change following Roux-en-Y surgery.

Overall, these results would suggest that many of the hepatokine, adipokine and myokine homeostatic parameters differ compared to individuals with normal BMI; however, it is unclear if these parameters are abnormal because the subjects still have class 1 obesity with a BMI of 34 kg/m^2^, or whether this represents a new normal homeostatic setpoint for these individuals as their clinical features have improved. Unfortunately, there was not a cohort of obese patients who achieved a BMI of 25 kg/m^2^ or less to answer this question. Therefore, post-surgical obesity patients may have “obesity memory” (metabolic hysteresis) (39) where the BMI of 34 kg/m^2^ is the new “normal” threshold that may not regress to that of subjects with a BMI of 25 kg/m^2^ or less who have never been obese.

A strength was that a single surgical procedure was used for the study; however, we do not know if differing bariatric surgery procedures would have a greater or lesser effect on these body homeostasis parameters. Additional strengths of this study include the long follow-up duration of 12-years for the population of the surgical and non-surgical comparator-subjects-with-obesity and T2D, and the comparison of normal homeostatic parameters in normal-weight subjects. However, further studies are needed to see when the body homeostasis markers return to normal, perhaps with a BMI between 26-29 kg/m^2^ i.e., overweight but not obese. As no subjects achieved absolute normalisation of their BMI, a sub-analysis of such a group could not be undertaken. A further limitation of the study was that the normal-weight subjects were 10 years younger, which may have had an impact despite adjusting for this statistically; thus, the normal-weight parameters are indicative and, ideally, a post-bariatric study population with subjects attaining a BMI of 25 kg/m^2^ or less would be needed for validation of the results. Additional limitations include the lack of baseline values of adipokines, hepatokines and myokines. In addition, data on diet and physical activity for the study cohorts was not analyzed between baseline and 12-years, though were comparable at baseline between the surgical and non-surgical patients. It is well recognized that diet and exercise may affect homeostatic parameters (40–42); however, bariatric surgery induces weight loss, forces dietary changes and allows increased activity and, therefore, it is difficult to adjust the model to account for each parameter. Whilst medication may affect the homeostatic parameters (43), we did include antidiabetic medication use as a covariate in the statistical models that did not change the conclusions, as the covariate was not significant in any model. A BMI-matched population to the post-surgical group appears attractive but would not be comparable in this study as those subjects would not have had the impact of weight loss and thus would not have been comparable.

In conclusion, bariatric surgery markedly improved anthropometric and metabolic features versus comparator-subjects-with-obesity at 12-year follow-up, indicating the benefit of weight loss. However, despite weight loss, these patients still had class-1 obesity, as reflected in the adipokine, hepatokine and myokine markers of body homeostasis that did not completely normalize to indicative values of normal-weight subjects, suggesting either that this is the new normal for these patients or that weight loss to a BMI <34 kg/m^2^ is needed for normalization of these parameters.

## Data availability statement

The raw data supporting the conclusions of this article will be made available by the authors, without undue reservation.

## Ethics statement

All procedures performed in studies involving human participants were in accordance with the ethical standards of the University of Utah’s Institutional Review Board (IRB_00006824) and with the 1964 Helsinki declaration and its later amendments or comparable ethical standards.

## Author contributions

AB, MR, SCH and SLA wrote the manuscript. AB, MR, SH and SA edited the manuscript. SCH performed the statistical analyses. AM contributed to data interpretation and visualization. Grant funding was awarded to SH. SA, AB, SCH, AM and MR contributed to study design and data interpretation. All authors contributed to the article and approved the submitted version. AB is the guarantor of this work.
